# *Pseudomonas aeruginosa* Diversification during Infection Development in Cystic Fibrosis Lungs—A Review

**DOI:** 10.3390/pathogens3030680

**Published:** 2014-08-18

**Authors:** Ana Margarida Sousa, Maria Olívia Pereira

**Affiliations:** CEB—Centre of Biological Engineering, LIBRO—Laboratório de Investigação em Biofilmes Rosário Oliveira, University of Minho, Campus de Gualtar, 4710-057 Braga, Portugal; E-Mail: anamargaridasousa@deb.uminho.pt

**Keywords:** *Pseudomonas aeruginosa*, cystic fibrosis, clonal diversification, phenotypic variation, mucoid phenotype

## Abstract

*Pseudomonas aeruginosa* is the most prevalent pathogen of cystic fibrosis (CF) lung disease. Its long persistence in CF airways is associated with sophisticated mechanisms of adaptation, including biofilm formation, resistance to antibiotics, hypermutability and customized pathogenicity in which virulence factors are expressed according the infection stage. CF adaptation is triggered by high selective pressure of inflamed CF lungs and by antibiotic treatments. Bacteria undergo genetic, phenotypic, and physiological variations that are fastened by the repeating interplay of mutation and selection. During CF infection development, *P. aeruginosa* gradually shifts from an acute virulent pathogen of early infection to a host-adapted pathogen of chronic infection. This paper reviews the most common changes undergone by *P. aeruginosa* at each stage of infection development in CF lungs. The comprehensive understanding of the adaptation process of *P. aeruginosa* may help to design more effective antimicrobial treatments and to identify new targets for future drugs to prevent the progression of infection to chronic stages.

## 1. Introduction

Cystic fibrosis (CF) is an autosomal recessive disease caused by a defect in the cystic fibrosis conductance regulator (CFTR) gene located in human on chromosome 7 that mainly affects lungs, digestive and reproductive systems, but also the secretory glands, such as the endocrine and sweat glands [1]. Although CF is a multi-system disorder causing several complications on the human body, its effects on lungs are the best studied so far due to the severe symptoms that patients suffer and high mortality rate associated to poor lung function.

It is generally accepted that CFTR acts as a channel that pumps chloride from the intracellular to extracellular space through the membrane of the epithelial cells that produce sputum. Several hypotheses have been formulated attempting to explain the relationship between CFTR deficiency and sputum accumulation. It has been considered that the transport of chloride partially controls water movement and consequently influences the production of thin and flowing sputum, fundamental to keeping the lungs protected [1,2]. The CFTR lacks causes, thus, a defective chloride secretion creating an osmotic gradient that, consequently, provokes water hyper-reabsorption and abnormal thick and sticky sputum [1,3]. This sputum with altered pH interfere with, reducing or even inhibiting the activity of epithelial antimicrobial molecules of innate immune system and ciliary functions, both crucial for homeostasis.

Other functions are also associated with CTFR, including inhibition of sodium absorption, of which loss causes excessive sodium (and water) absorption, regulation of HCO_3_^−^ and some proteins transport through epithelial cell membranes [3,4]. The relevance of the latter mechanisms in CF airway is unclear, however, it is believed that reduced chloride secretion or sodium hyper-absorption can occur. Both mechanisms lead to airway-surface-liquid depletion and sputum viscosity increase, causing impaired cilia beats and accumulation of thick dehydrated airway sputum, which profoundly accounts for the typical symptoms suffered by CF patients [4,5]. The defective mucociliary transport and the compromised immune defenses predispose CF patients to the establishment of recurrent bronchopulmonary infections. Sputum retention leads to infection and consequently to inflammation, and this circle perpetuates itself since inflammatory products, such as elastase released by neutrophils, stimulate sputum secretion and breakdown [3,6]. The accumulated sputum is rich in nutrients being, thus, a good environment for microbial colonization [7,8]. CF lungs are infected with a complex microbial flora, mainly composed by bacteria, provoking acute and chronic infections that result in decline of the lung function, respiratory failure, and premature death of patients. Once bacterial infections are established, their eradication by antibiotic treatment is hardly ever achieved [9,10].

Some progress was made in this field extending the life expectancy of CF patients, however, it remains very reduced, around 37 years, mainly because of bacterial infections [11]. In the last decades, new therapies have emerged, based on the knowledge of CFTR dysfunction and airway CF microbiome, such as targeting CFTR replacement, stimulation of alternative chloride channels, inhibition of sodium absorption, and airway rehydration, in order to avoid sputum accumulation and, consequently, the establishment of bacterial infections [3,4,5,12]. None of these strategies has sufficient potential to stop CF infections development thus far. The actual and more effective approach to fight CF-associated infections relies on antimicrobial treatment. Currently, there is no consensual antimicrobial treatment to eradicate bacterial infection from CF lungs [13,14]. Treatments vary among clinics, countries, and even continents. Numerous strategies have been used varying in route of antibiotic administration (systemic, oral, inhaled antibiotics, or routes combination), classes of antibiotics, and treatment duration. Inhaled antibiotics, mainly aminoglycosides, have high success rates in bacteria eradication, in particular against *Pseudomonas aeruginosa*, due to the direct delivery of high-dose of antibiotic to the bronchial lumen space with limited systemic toxicity. For instance, a tobramycin inhalation solution has been used to treat long-term and chronic bacterial infection with significant benefits for lung function delaying re-infection and reduce mortality [15,16,17]. Oral and intravenous antibiotics have also attracted interest and currently quinolones, in particular ciprofloxacin, are the most used. However, ciprofloxacin usage is somewhat limited due to the rapid emergence of resistance. As a solution, ciprofloxacin is frequently combined with other antibiotics through other routes of administration. Combination of inhaled colistin or inhaled tobramycin with oral ciprofloxacin has been used successfully [13]. Some authors had suggested the still used broad-spectrum penicillins and cephalosporins in efforts to improve outcomes for CF patients infected with *P. aeruginosa* [18].

Other antibiotics have been introduced and used as alternative agents, such as inhaled amikacin, aztreonam lysine, and the combination of fosfomycin and tobramycin [12,13,14]. However, it has assisted to the failure of these antibiotic courses, making urgent the comprehension of the mechanisms underlying antibiotic resistance to rapidly define effective strategies to eradicate those infections.

## 2. Pseudomonas aeruginosa

The microbial community resident in CF lungs is known to be complex and it has considerably changed, mainly due to alterations in antibiotic regimens. Nevertheless, *P. aeruginosa* is still the most common pathogen isolated from CF sputum, being more prevalent in adults [2,10,19].

*P. aeruginosa* is a versatile microorganism, ubiquitously distributed in different environments, including terrestrial, aquatic, animal, human, and plant. It is a Gram-negative opportunist pathogen in hospitalized or immune-compromised patients, causing infections, such as pneumonia, burn, wound, urinary tract and gastrointestinal infections, otitis media, and keratitis [19,20]. Its versatility arises from its large genome, with nearly 6000 genes that enclose, for instance, genes associated with diverse metabolic pathways, virulence factors, transport, efflux, and chemotaxis, conferring to *P. aeruginosa* great adaptive ability. Moreover, this bacterium is able to coordinate metabolic pathways, optimize nutritional and reproductive potential according to the surrounding conditions and resources and, thus, it can survive, grow and cause infection in different environments [20,21].

The presence of *P. aeruginosa* in CF airways is highly associated with poor lung function, morbidity and mortality of patients. Despite the inflammatory response and the long-term and intensive antibiotic treatments, infections caused by *P. aeruginosa* persist in CF lungs. Once entering in CF airways, *P. aeruginosa* is virtually impossible to eradicate due to its remarkable genome plasticity that allows it to rapidly adapt to the greatly stressful CF environment [2,22,23]. After *P. aeruginosa* colonization, patients may suffer of successive episodes of re-colonization until resulting in a chronic infection that can persist from years to decades, or even never eradicated [23,24]. Several factors can influence the infection course in CF airways and, unfortunately, there is limited knowledge about the characteristics of this microorganism that have impact on the severity of infection. Until now, it is just known that during CF infection development, *P. aeruginosa* switch from an acute environment virulent pathogen, characteristic from early infection stages, to a CF-adapted pathogen, typical of chronic infection stages [6,21,25]. This review aimed to provide an overview of the successive adaptations that *P. aeruginosa* undergo, and to describe their impact on long-term persistence in the airways. The identification of the genetic background, interactions, and strategies, used by *P. aeruginosa* are crucial, and a prerequisite to develop new approaches for effectively eradicate lung infections.

## 3. Sources of Phenotypic Diversification

**Figure 1:**
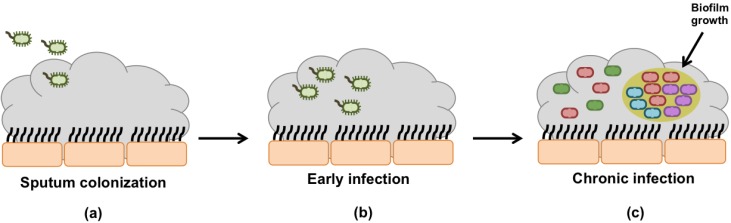
Time course of *P. aeruginosa* infection development. (**a**) Sputum colonization stage - *P. aeruginosa* equipped with full virulence factors enter in CF sputum; (**b**) Early infection stage—*P. aeruginosa*, which exhibit the environmental or wild-phenotypes species characteristics, starts its adaptation to CF environmental conditions; (**c**) Chronic infection stage—*P. aeruginosa* is full adapted to CF environment. At this stage, there is high phenotypic and genotypic diversity and formation of biofilms.

The long-term persistence of *P. aeruginosa* infections in CF lung is associated with clonal diversification, or expansion, into specialized phenotypes (Figure 1). Driven by the challenging selective pressures imposed by the typical CF conditions, e.g., interspecies competition, deficient oxygen availability, biofilm growth, the immune system action, oxidative stress, and antibiotic treatment, *P. aeruginosa* progressively generates phenotypes specially adapted to CF airways conditions [2,9,26,27]. The CF selection forces are evident when clinical isolates of *P. aeruginosa* are frequently mucoid and highly resistant to antibiotics. Indeed, mucoid variants are rarely isolated from non-CF environments, suggesting the existence of specific CF selective pressure [2,23]. For this reason, *P. aeruginosa* conversion from non- to mucoid form is considered the hallmark of CF airway.

However, other phenotypic features of clonal variants adapted to CF airways have been frequently observed, including small colony variants (SCV), non-pigmented variants, increased antibiotic resistance, altered metabolic pathways, and attenuated virulence potential [3,22,27]. This phenotypic variation occurs for adaptation to the different niches in CF airways. The repeated occurrence of these particular phenotypic features and/or gene expression in chronic isolates, sampled from different patients and clinical settings, suggests the existence of a parallel evolution of *P. aeruginosa* in CF airways [28,29]. This topic is discussed in detail in Section 4.2.

The biofilm-lifestyle represents a reservoir of high phenotypic diversity and it is considered one of the most important adaptive mechanisms of *P. aeruginosa* within CF sputum (Figure 1c) [22,30,31]. Worlitzsch *et al.* (2002) [32] have shown that in the conductive zone, the region from the trachea to the terminal bronchiolus, *P. aeruginosa* grows mainly in biofilms, whereas very few bacteria are localized at the epithelial surface of the bronchi. Biofilms are microbial communities encased in self-produced matrix composed of exopolysaccharides, proteins and DNA [33,34,35]. Nowadays, biofilms are recognized as an important issue in human disease management due to their notoriously resistance, achieving 10- to 1000-fold higher tolerance to antimicrobial agents than corresponding planktonic bacteria [35,36]. Biofilm resistance has multifactorial nature resulting from the combination of several mechanisms, including restricted penetration of antimicrobials through the exopolysaccharide matrix, slow growth of bacteria within biofilms caused by nutrient and oxygen restriction, and accumulated metabolic wastes, and quorum-sensing (QS) molecules [37,38,39,40]. The limited penetration of antibiotics and immune defenses through the exopolysaccharide matrix is definitely a great contributor for their ineffective action and *P. aeruginosa* persistence*.* Alginate is the major component of CF biofilm matrix providing structure and protection to bacteria from the stressful environmental conditions of CF lungs. Augmented levels of alginate are generally observed in CF patients chronically infected and it is associated with poor prognosis because alginate triggers a vigorous antibody response [41,42].

Airway CF biofilms are genetic, proteomic and physiologic different of surface-attached biofilms formed, for instance, on indwelling devices (catheters, prostheses, pacemakers, stents), and medical and clinical equipment. Instead of the direct surface colonization, clearly observed in device-associated infections, bacteria in the CF lungs preferentially form multicellular clusters or macrocolonies within the sputum and not on the surface epithelium in the bronchi and non-respiratory bronchioles as initially supposed [31,32,43,44]. Additionally, the environment in which CF-associated biofilms are formed is considered to be microaerophilic or anaerobic. Bacteria enter and colonize CF sputum, consume oxygen via respiration, and generate steep oxygen gradients within the sputum [9,30,44]. The limited oxygen availability to potentially anaerobic environments in CF sputum was confirmed by direct *in situ* oxygen measurements using a microelectrode [32]. The oxygen-limited and anaerobic growth conditions significantly increase antibiotic resistance of biofilm-forming bacteria [45].

Until now, it is not clear what time bacteria after CF airway colonization switch to sessile lifestyle, but it is known that biofilm formation enables bacteria to successfully establish chronic infections. Presumably, *P. aeruginosa* form biofilms in response to stressful conditions including microaerophily and/or antibiotic treatments [23,46].

To switch from planktonic to biofilm mode of growth, bacteria undergo a number of complex physiological, metabolic, and phenotypic differentiations. For instance, biofilm-growing bacteria undertake specific changes in protein regulation, especially those related with proteins involved in resistance to oxidative damage, exopolysaccharide production, phospholipid synthesis, and membrane transport [47,48,49]. Global gene expression analyses of mature *P. aeruginosa* biofilms have revealed 1% of differential gene expression between the planktonic and biofilm mode of growth, with 0.5% of the genes being activated and about 0.5% being repressed [50]. Among the transcription factors, repression of flagellar and pili genes and stress response regulator genes, such as *rpoS*, hyperexpression of genes for ribosomal proteins and metabolism and transport functions were the most identified. Interestingly, in the same study, QS-regulated genes were not identified. QS is a cell-cell communication system used by bacteria to regulate gene expression in response to fluctuations in cell-population density and it has being reported to play a role in early and later stages of biofilm development. *P. aeruginosa* has two distinct QS systems, termed las and rhl [51,52]. The lack of las QS system allowed the formation of biofilms, however, does not allow them to achieve the mature stage. The *rhl* QS system has been reported as active in the early stages of biofilm development and its block may prevent biofilm formation [47,53]. Other regulatory systems can influence early stages of biofilm development, such as the global virulence regulator GacA [54], the catabolite repression control protein Crc [55], and the response regulator proteins AlgR [56]. The blockage by mutation of those factors has demonstrated a significant decreased of biofilm formation.

Gene expression may vary during biofilm development, which means that there is a stage-specific temporal and spatial gene expression patterns. This is particular relevant concerning the resistance of mature biofilms to antimicrobial treatment. The biofilm-specific phenotype can trigger mechanisms responsible for antimicrobial resistance and persistence and consequently enhanced pathogenicity. *P. aeruginosa* genome sequencing have revealed that a mature biofilm can express several cluster genes encoding efflux pump involved in resistance to some antibiotics [50].

The great variability or heterogeneity of phenotypes included and developed within biofilms is certainly one of the major contributors for sessile bacteria recalcitrance that it is not observed in planktonic state [57,58,59]. Within biofilms, various heterogeneous environments exist as a result of the distinct levels of nutrients and oxygen availability and accumulated metabolic wastes that bacteria have to face and adapt in a process similar to CF airway adaptation [60]. This range of microniches with specific biological activities may somewhat be translated by the several distinct colony morphologies that biofilm-growing bacteria adopt when grown in a solid media. Such trait diversification profits the whole population, with diverse abilities to face environmental challenges, as long as bacteria coordinate with each other. Bacterial cooperation and differentiation is facilitated through the production and perception of QS small signaling molecules called autoinducers. This interbacterial communication is mediated by two types of molecules, *N*-acylhomoserine lactones (AHL) and 4-quinolones, allowing bacteria to perceive their density and regulate their gene expressions properly. For instance, up-regulating genes encoding virulence factors such as those related to the production of enzymes or toxins, optimizes the metabolic and behavioral activities of bacteria within the community [61,62].

Biofilm heterogeneity is also reflected in distinct antibiotic susceptibility profiles. Due to the different biofilm-cell physiological states, biofilms have typically a top-to-bottom decreasing susceptibility profile. Antibiotics are effective against the cells located in the top of the biofilm, generally in active state, in contrast to the middle and bottom zones, in which cells have reduced or even an absence of metabolic activity. Even when antibiotics reach the middle and/or bottom biofilm zones, the majority of them have no activity against dormant cells and, thus, are unsuccessful in biofilm eradication [39,60,63].

Planktonic *P. aeruginosa* cells are also found in CF sputum [31]. Due to alterations in CF environment, such as pH and oxygen and nutrients availability, biofilm-cells dispersion may occur [64]. The dispersal of biofilm population provides to *P. aeruginosa* an opportunity to colonize new zones or niches and, thus, perpetuate infection. In fact, dispersal events can be responsible for the acute exacerbations observed in chronic infections [46,58,65].

The whole adaptation process to CF airways can be accelerated by the emergence of mutator phenotypes (or hypermutable phenotypes) which have high mutation rates up to 1000–fold than non-mutator phenotypes [66,67,68]. In extreme selective conditions, such as those occurring in CF airways, this sophisticated mechanism improves the microevolution of *P. aeruginosa* accelerating its intraclonal diversification. The emergence of phenotypic variants and mutators can be intrinsic, relying on mutations (or recombinations) caused by defects on one of the several DNA repair or error avoidance systems, combined, or not, with extrinsic or environmental factors, such as competition for different niches in a spatially heterogeneous environment as CF airways, and/or selection that favors any mutant as a better “fitter” to CF airways [67,69,70]. Mutators can also be stimulated by environmental factors, such as the presence of reactive oxygen species (ROS) generated from inflammatory responses [71]. ROS can trigger the generation of phenotypic variants damaging DNA and cause mutations in bacteria. Further, sub-inhibitory or sub-lethal concentrations of antibiotics can induce mutations and recombinations and, consequently, supporting the emergence of phenotypic variants and mutators [26,67,72]. The genes mainly affected are the antimutator genes *mutS*, *mutL*, and *uvrD* but it can be observed defects, as well in the genes *mutT*, *mutM*, and *mutY* [66,73,74].

The amount of mutators in biofilms is significantly higher than in planktonic state. This condition may explain why biofilm-associated bacteria exhibited enhanced antibiotic resistance, and frequently multidrug resistant, and high genetic diversity leading the emergence of diverse phenotypic variants [75,76].

The generation of various subclonal variants represents a huge biological advantage because it prepares the *P. aeruginosa* population for extreme and unpredictable stresses (insurance hypothesis) supporting the long-term survival of this pathogen [59,77]. Mutators achieve more quickly CF adaptation due to the expression of virulence traits, antibiotic resistance, metabolic functions, and increased ability to form biofilms, all these features representing a serious clinical problem [67,78,79]. In effect, mutators can increase the transcription of genes involved in the metabolism of fatty acids and amino acids crucial for obtaining energy in CF ecological niches where aerobic respiration is not possible [80]. On the other hand, mutators may have a reduced ability to survive in other distinct environments, indicating they can reach high levels of habitat- or niche-specialization spending their biological fitness [23,67]. During infection development non- and mutators coexist in CF airways, however, mutators prevail at chronic stage, which may be indicative that they have an adaptive advantage.

The combined action of all these sources of clonal diversification may achieve impressive levels of diversification, adaptation, and evolution, promoting the persistence of the bacterial populations in CF airways. Therefore, these sources should be intensively studied in order to understand the underlying mechanisms to further block them and combat the recalcitrant infections.

## 4. *P. aeruginosa* Evolution and Adaptation during Infection Development

The regular sampling of CF sputum has allowed performing a detailed characterization of *P. aeruginosa* over infection development through DNA sequencing and other approaches, such as transcriptomic, metabolomics and proteomic techniques. Therefore, it is now possible to start drawing an evolutionary trajectory of *P. aeruginosa* within CF airways.

**Figure 2:**
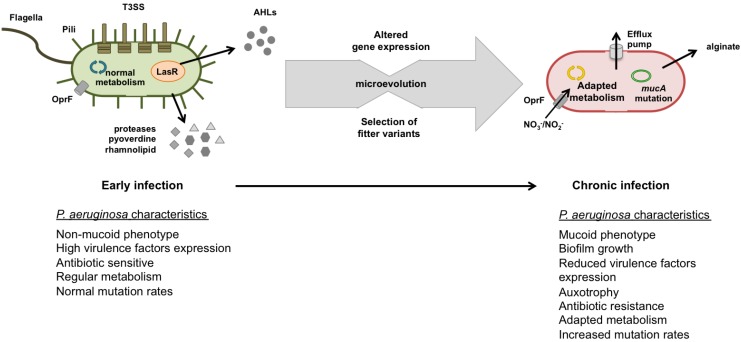
Representation of *P. aeruginosa* microevolution during infection in CF airways. At early stage of infection, *P. aeruginosa* is full equipped with cell-associated virulence factors, including flagella, pili, type 3 secretion systems (T3SS) and secreted virulence factors (e.g., proteases, pyoverdine, and rhamnolipid) and exhibit antibiotic sensitivity. At the chronic stage of infection, *P. aeruginosa* is fully adapted to CF environment and exhibits a variety of adaptations, including overproduction of alginate, loss of the implicated virulence factors for initial infection establishment, are resistant to antibiotics (expression of efflux pumps), and adapted metabolism. This microevolution occurs by the repeated interplay of mutation and selection.

During infection development genotypes and phenotypes differ markedly from those that initially colonized CF airways (Figure 2). Microbiological studies have reported changes in *P. aeruginosa* phenotypic and genetic traits, relevant in the context of bacterial pathogenesis, and different antibiotic resistance patterns along infection development, as well as after antibiotic treatments. Similar evolution and adaptation profiles were observed in distinct clonal linages of CF-adapted strains, suggesting that, in fact, there is a similar selective pressure in CF airways. This evolution and adaptation processes lead to the generation of several phenotypes varying in characteristics, such as colony morphology with distinct consistency, size, texture and color, the inactivation of QS, hypermutation, loss of the O-antigen components of the lipopolysaccharide (LPS), loss of motility, resistance to antibiotics, changes in nutritional requirements, and other virulence-associated traits [2,26,81,82]. In fact, some of those factors have been considered the hallmark of CF disease and can determine the infection stage, such as the conversion of *P. aeruginosa* to mucoid phenotype, loss of motility, and the emergence of SCV. However, many other characteristics have been described across all phenotypes isolated so far.

Antibiotics have provided significant control of bacterial infections in CF airways, however, the occurrence of antibiotic resistance and the lack of new drugs or therapeutic strategies make imperative the identification of alternative targets for treatment. For understanding the mechanisms underlying bacterial adaptation to CF environment and the resistance to antibiotic treatments, an overall picture of the actual knowledge about the *P. aeruginosa* populations, resident in CF lungs, is needed.

The compilation of the phenotypic traits exhibited by bacteria according the infection stage is a hard task due to the lack of agreement on the definitions of early, intermediate, and chronic colonization and infection stages. In this paper, the evolution, adaptation, and diversification profiles of *P. aeruginosa* were reviewed and compared agreed by the “European Consensus” that just considers two infection stages, early and chronic stages, according to the presence of *P. aeruginosa* being lower or higher than six months, respectively [83].

### 4.1. Early Infection

Most CF patients acquired pathogens mainly from environment, especially in clinical settings where CF patients remain for long periods of time. For this reason, respiratory infections associated to CF patients can be in somewhat considered nosocomial infections. As CF patients acquired environmental pathogens, early CF isolates exhibited identical microbial characteristics of their environmental or wild-phenotypes species (Figure 2) [21,84,85]. At the first colonization of CF airways, *P. aeruginosa* have to regulate properly its gene expression to quickly adapt to this new environment, including host immune defenses, antibiotics, and different substrate composition.

The bacterial characteristics among CF acute isolates significantly vary, however, there is a trend towards high virulence potential and cytotoxicity and lower frequency of mutators strains [86]. The expression of virulence factors, including cell-associated and secreted virulence factors, is considered to be fundamental at early stage for the success of infection establishment. For instance, (i) the increased production of pyoverdine, haemolysin, and phospholipase C; (ii) the augmented production of rhamnolipid, regulated by QS, helps biofilm formation that protects cells against oxidative stress, decreases liquid surface tension, due to its biosurfactant feature, and facilitates the access to nutrients within biofilms; (iii) the increased production of total protease that promotes mucoidy essential for long-term bacterial persistence; (iv) the swimming and twitching motilities; and (v) the expression of the type III secretion system that augments cell cytotoxicity potential and facilitates infection development [23,84,85,87,88].

Typically, *P. aeruginosa* exhibited a non-mucoid phenotype, sensibility to antibiotics and have low bacterial density in lungs in contrast to chronic infections [24,85]. Acute CF isolates produced AHL suggesting that QS circuit plays a role for *P. aeruginosa* pathogenesis at this stage of infection. Afterwards, QS seems be no longer needed and *lasR* mutants are frequently isolated. Mutator strains are not prevalent at this stage because they are not efficient to establish a primary infection [51,89].

At the early stage, eradication is still possible whether an antibiotic treatment was started as soon as possible. Otherwise, 20% of those first *P. aeruginosa* colonisations could become directly chronic infections and may persist up to the end of patient life [24,85]. Following *P. aeruginosa* eradication, it is common a new acquisition event with a different genotype or a re-colonization with the same genotype. Re-colonization with the same genotype may occur due to the persistence of the environmental source or due to the colonization of the upper airways, such as the paranasal sinuses [90,91]. Upper airways can function as reservoirs of pathogens, and interchange of *P. aeruginosa* can be possible. Colonization of the CF airways with mucoid strains is associated with an accelerated rate of decline in pulmonary function, however, there is some evidence that early acquisition of mucoid strains could be successfully achieved [24,92].

In summary, although the virulence potential of early CF isolates is higher than chronic isolates, they exhibited increased antibiotic sensitivity. Therefore, *P. aeruginosa* early detection and eradication are currently the main goal to avoid infection progression to chronic stage. Early infections are intensively treated with antimicrobial therapy resulting in *P. aeruginosa* eradication, at least temporal eradication in the majority of patients [24]. In cases of antimicrobial therapy failure, infection can shortly evolve to chronic infection. Identification of the CF patients who may evolve to chronic infections based on the acute bacterial characteristics is still not possible because of factors related to host-pathogen and pathogen-pathogen interactions may play a role and their impact is, thus far, unknown [86,93].

### 4.2. Chronic Infection

The continuous and selective pressure over the population leads to the emergence of diverse phenotypic and genetic variants specially adapted to CF airways. It has been observed, among chronic *P. aeruginosa* isolates, that there are alterations in colony morphology, namely the conversion to the mucoid morphotype, to SCV and non-pigmented variants, changes in surface antigens, lack of some virulence factors expression, increased antibiotic resistance, overproduction of exopolysaccharides, and modulation of microaerobic and anaerobic metabolic pathways (Figure 2). These alterations suggest a survival strategy to switch off or, at least, to reduce the expression of some traditional virulence factors. In fact, this *P. aeruginosa* strategy consists in saving or reducing energy costs with virulence factors expression in favor of alternative metabolic pathways crucial at this stage.

The repeated occurrence of these phenotypic features in chronic isolates of *P. aeruginosa* indicates that they may be a result of parallel evolution, which means that related microorganisms develop the same adaptive features in identical, but independent, environments [28]. Several transcriptomic studies have profiled isolates of *P. aeruginosa* in attempt to find the common route towards the chronic phenotype and the mechanisms underlying such a route. Longitudinal studies using transcriptomic approaches have provided relevant information regarding the genetic changes undergo by *P. aeruginosa* and allowed comparing the expression of specific set of genes among patients in different periods of time. Gene expression changes in multidrug efflux pumps and regulators of quorum sensing and alginate biosynthesis have been identified, being the two latter hotspots of mutations [21,28,94,95].

In the scope of genomic evolution in chronic CF lung infection, it should be highlighted that the notorious work performed by the Copenhagen and Hanover clinics, which had regularly collected *P. aeruginosa* from the CF lungs of all their patients in the 1970s and 1980s, and performed the genome sequencing of the isolates. They started their investigation with the most prevalent clones, the C and PA14, and observed that both clones convert their phenotype becoming deficient in the LPS O-antigen, with impaired motility and decreased siderophores secretion, as well as in other virulence factors expression and remaining non-mucoid [96]. Those isolates later collected just exhibited impaired competitive growth. These cases demonstrated that the evolutionary transition might be through the additive effects of various mutations. However, a single loss-of-function mutation can induce dramatic changes in *P. aeruginosa*, as those observed through the mucoid variant, due to the pleiotropic effects of *mucA* mutation [2,21].

Mucoid colony morphology results from alginate overproduction, absence of flagellin and pilin and expression of other virulence factors. Within the mucoid form, *P. aeruginosa* is more difficult to eradicate because it is highly resistance to antibiotics, as well as to the actions of host immune defenses, for instance, to phagocytosis, mediated by macrophages and neutrophils and to antibodies oponization [3,30,97]. Alginate overproduction is on the basis of such protection and resistance. Alginate promotes *P. aeruginosa* encapsulation and biofilm formation protecting sessile bacteria from the action of ROS, antibiotics and host immune defenses persisting in CF lungs [22,23]. Because mucoid *P. aeruginosa* raise a vigorous antibody response, its presence contributes to tissue damage, decreased lung function, and a decline in health [41,42]. The genetic mechanisms underlying *P. aeruginosa* transition to the mucoid form have been intensively studied and conversion is mainly caused by mutational inactivation of the *mucA* gene and rarely of *mucB* or *mucD* genes [98,99]. *mucA* gene encodes a cytoplasmatic membrane bound protein that acts as anti-σ-factor, σ^22^, limiting the expression of the *algD* operon required for alginate synthesis. MucA binds to AlgT (also termed AlgU) that negatively controls the transcription of the *algD* gene. Inactivation of *mucA* results in upregulation of AlgT and production of alginate [2,100,101]. In fact, σ^22^ can also activate the transcription of several other genes related to virulence factor expression and to stress response, including heat shock, and osmotic and oxidative stress [21,102]. Additionally, it can repress the expression of type III secretion system (T3SS) genes through activation of AlgU that actives the regulatory genes *algP*, *algQ*, *algB*, and *algR*. AlgR, a global regulator, affects the expression of multiple genes including T3SS [103]. This suggests an impressive coordination of two high-cost energy systems in order to bacteria persist in CF airways.

Although mucoid phenotype is very successful at chronic infection stage, non- and mucoid phenotypes can coexist [31]. Non-mucoid isolates can occur from persistence of *P. aeruginosa* wild-type or re-conversion of mucoid phenotypes (revertants). Mucoid phenotypes can revert to non-mucoid form in the absence of *in vitro* selective pressure or through secondary mutations. Non-mucoid phenotypes can also carry *mucA* mutation, suggesting that mutation occurred when selective pressure occurs and when its vanished secondary mutation takes place [21,104]. This suggests that the production of alginate represents a high-energy cost and, thus, its unstable feature. At this stage, non-mucoid phenotypes have its alginate production at minimal levels [52].

The conversion to mucoid phenotype also promotes the biofilm mode of growth. The presence of biofilms is a key factor for the persistence of infection in CF airways. Biofilm-cell differentiation and dispersal events contribute to the generation of higher diversity that consequently increases the ability of *P. aeruginosa* to colonize new niches in CF airways, thus, perpetuating infection [46,58,59].

Another variant frequently isolated from chronic CF lung infections are the SCV. SCV designation comes from their small-colony size, typically 1–3 mm after 24–48 h of growth on agar media [105]. SCV are normally hyperpiliated, hyperadherent, excellent biofilm formers, and exhibit autoaggregative behavior and increased twitching motility [106,107,108,109]. In addition, SCV display augmented resistance to several classes of antibiotics, notably to aminoglycosides, contributing to their persistence in CF airways and poor lung function. SCV are generally selected after prolong antibiotic treatments [105,110]. In contrast with the mucoid phenotype, the mutations that arise in SCV appear to be very diverse and a challenge for the understanding of the underlying molecular mechanisms [111]. This phenotype may arise from the increased expression of the *pel* and *psl* polysaccharide gene loci and elevated intracellular c-di-GMP levels that enhance the ability to form biofilms, motility, and the expression of the type 3 secretion system, persisting, thus, more efficiently in the CF airways [109,111]. Until now, SCV were mostly studied regarding *Staphylococcus aureus* but, currently, it has been equally assumed that *P. aeruginosa* SCV is, as well, a cause of infection persistence [35,112,113,114].

Other colony morphologies have been isolated from CF airways typically exhibiting rough texture due to alteration of the lipid A moiety of LPS. Those variants contain a few, short, or no O side chains and exhibited augmented proinflammatory activity [23].

In chronic infections, CF isolates typically exhibited impaired motility, namely swimming and twitching, due to the absence of flagella and pili, respectively. Lacking flagella (e.g., *fliC* mutant), *P. aeruginosa* isolates are hardly phagocytosed by alveolar macrophages and neutrophils helping to evade the host immune defenses, allowing its persistence in CF airways [23,52]. Moreover, *P. aeruginosa* lives in this chronic stage in biofilm-growth mode in which cells downregulate flagellum and type IV pili since they are no longer needed to move across sputum and along epithelial cell surfaces [115]. Nonpiliation may arise from mutations of *pilB*, encoding an ATPase needed for the extension and retraction of pili, or defects in *pilQ* gene, required to extrude the pilus through the bacterial outer membrane [116]. Nevertheless, the majority of CF isolates exhibited *rpoN* mutations that provoke the loss of both pili and flagella [117].

Chronic CF isolates show other attenuated virulence factors such as reduced production of AHL, proteases, phospholipase C, loss of pyoverdine, pyocyanin, and elastase and decreased cytotoxicity potential, due to the switching off of the T3SS. These alterations reduce the efficacy of the immune system to recognize *P. aeruginosa* helping, thus, its persistence in CF airways [23,52].

Chronic *P. aeruginosa* isolates are commonly *lasR* mutants. *lasR* gene encodes QS transcriptional regulator LasR and its downregulation may explain the reduced or absent production of AHL at this infection stage, the autolysis and the iridescent gloss of *P. aeruginosa* colonies, the growth advantage on amino acids and decreased virulence potential [94,118]. In addition, *lasR* mutants can use nitrate (NO_3_^−^) and nitrite (NO_2_^−^) as the terminal acceptor of electrons allowing *P. aeruginosa* growth in anaerobic niches. The loss of social and cooperative behavior may confer an adaptive advantage since the production of QS signal molecules, such as *N*-(3-oxo-dodecanoyl)-l-homoserine lactone (3-oxo-C12-HSL), *N*-butanoyl-l-homoserine lactone (C4-HSL), 2-heptyl-4(1H)-quinolone (C7-HHQ), and 2-heptyl-3-hydroxy-4(1H)-quinolone (PQS), are costly. Avoiding these costs, *P. aeruginosa* can ensure its persistence for the long-term [52,81]. QS seems just contribute to *P. aeruginosa* pathogenesis at colonization or acute stages [81,119].

To survive and adapt to CF airways, *P. aeruginosa* has, as well, to adapt its metabolic pathways. In fact, those alterations are also considered a marker of the chronic stage. The generation of energy is mainly based on oxidative substrate catabolism, however, *P. aeruginosa* is able to use alternative electron acceptors. The carbon metabolism of *P. aeruginosa* is mediated by catabolite repression control, which determines the catabolism of substrates in a preferred order. Short-chain fatty acids, amino acids, and polyamines are generally the preferred carbon sources and sugars the less favored [120].

CF sputum contains high amount of mucin, DNA, lipids, amino acids, and proteins that *P. aeruginosa* can uptake. Several studies have reported that peptides, amino acids, and fatty acids belonging to host defenses, such as prostaglandins and phosphatidylcholine, supports *P. aeruginosa* growth in CF airways [7,80]. The increased availability of those components is highlighted by the frequent isolation of auxotrophic variants for different amino acids, however the adaptive advantage of those variants in CF airways is unclear thus far [121]. Arginine and methionine are the most common auxotrophisms detected among CF isolates [122,123,124]. Auxotrophic variants may be more common than actually reported because those variants may be less cultivable *in vitro* and, consequently, under-estimated.

As mentioned earlier, the distinct oxygen availability in CF sputum represents a challenge for *P. aeruginosa*, which undergoes metabolic and physiologic changes with a high impact on antibiotic treatments. Along chronic infection progress, *P. aeruginosa* can face aerobic, microaerophilic, and anaerobic zones within the CF sputum and different enzymes, transporters, and regulators for different metabolic pathways are up-regulated to achieve this adaptation [86]. *P. aeruginosa* preferentially uses oxygen as terminal electron acceptor to obtain maximum energy. However, *P. aeruginosa* is able as well to survive and growth in hypoxic and anoxic CF niches adapting its metabolic pathways. Under anaerobic conditions, *P. aeruginosa* can obtain energy to grow from the denitrification or fermentation of arginine [30,80]. Denitrification or anaerobic respiration allows the detoxification of NO, generated during infection development. The outer membrane protein, OprF, represents a crucial factor in anaerobic metabolism since it allows the permeation of the ions NO_3_^−^/NO_2_^−^ fundamental to perform denitrification [30,44,80]. In niches where oxygen and N-oxides are unavailable, but amino acids are in high amounts, *P. aeruginosa* can use fermentation of arginine, converting it into ornithine [80]. In cases of arginine limitation, *P. aeruginosa* can still convert pyruvate into acetate and, thus, obtain energy. In this way, anaerobic biofilms can be formed and support *P. aeruginosa* survival, growth, and persistence in CF airways. Anaerobic environments increase *P. aeruginosa* antibiotic tolerance and the robustness of biofilms through the increased production of alginate, typically via mutation in *algT*/*algU* [45,125,126,127]. Consequently, CF mucoid strains, that are alginate producers, are selected at this chronic stage. Despite all these findings about the metabolic pathways used by *P. aeruginosa* during chronic infections, information about the regulation and the mechanisms underlying each metabolic pathway and the specific effects on virulence, antibiotic resistance and persistence in CF lungs is still scarce. Certainly, the understanding of those mechanisms could help new therapeutic solutions to arise.

The presence of mutators within the populations are characteristic of chronic infections, considered a virulence determinant of *P. aeruginosa* and often associated with parallel occurrence of subpopulations with distinct phenotypic characteristics [66]. Mutators ensure *P. aeruginosa* survival against various CF stress conditions and other unpredictable stress factors, and being, moreover, a key factor in the development of multi-antimicrobial resistance [73]. At the chronic stage, hypermutability increases, also due to the presence of biofilms, in which the frequency of mutators is higher than the free-living mode of growth [75,128]. The transcriptome comparison of a non- and mutator revealed significantly transcriptional changes among them. In fact, it was observed that mutators exhibited increased levels of genes involved in amino acid and fatty acid metabolism [80].

Chronic infections are usually punctuated with acute exacerbations in which *P. aeruginosa* may regain the increased levels of acute virulence of early stages, suggesting that the expression of some virulence factors can be reversible [65].

Despite the intensive and long antibiotic treatment, chronic infections of *P. aeruginosa* are rarely eradicated due to the occurrence of antibiotic resistance. It is frequently observed β-lactam-resistant *P. aeruginosa* phenotypes, due to the derepression of chromosomal b-lactamase [129], as well as ciprofloxacin [130], colistin [131], and tobramycin-resistant phenotypes, and even multi-drug resistance [132]. The main reasons for such increased antibiotic resistance is the biofilm-growth style and the presence of mutators [73,133,134,135,136].

In summary, the exhibition of certain characteristics, including alginate overproduction (mucoid phenotype), slow growth, alternative metabolic pathways, antibiotic resistance, and loss of virulence factors expression, is currently considered a chronic phenotypic profile and the end-point result of *P. aeruginosa* evolution in CF airways. *P. aeruginosa* clearly adopts a strategy aiming to reduce its energy costs in favor of activation of other biological pathways that ensure its long-term persistence. The actual evolutionary “model” of *P. aeruginosa* within CF airways consists in an initial and rapid adaptation period dominated by positive selection and adaptive mutations, followed by a period with minor phenotypic changes dominated by negative selection and fewer adaptive mutations [2]. This evolutionary process ends with an advent of a lineage of highly adapted bacteria with impressive ability to persist in CF lungs for long-term. Despite the assumption of parallel evolution to CF-well adapted phenotypes and the limited number of adaptive features, it is important to highlight that the actual evolutionary route towards a common profile among different patients is still not well understood. In fact, genomic and transcriptomic studies have just begun tracking *P. aeruginosa* evolution.

## 5. Conclusions

The study of the adaptation process and dynamical evolution of *P. aeruginosa* within CF lungs, and its impact on bacterial pathogenicity and virulence, is currently a topic of most importance in disease management. In this paper, the most common evolutionary profile of *P. aeruginosa* reported by researchers and clinicians were reviewed, however, other evolutionary, phenotypic, and genotypic profiles can be found in different demographic locations, clinics, and patients.

Longitudinal studies of clonal variants of different CF patients have tried to identify a common “expression signature” of *P. aeruginosa* over time. Genome sequencing, transcriptomic, and proteomic analysis have advanced the understanding of *P. aeruginosa* evolution, epidemiology and response to CF stress conditions, however there is still limited information about such “expression signature”. During adaptation, *P. aeruginosa* undergoes complex, structural and dynamic changes over the time. CF isolates from acute infections differs poorly from non-CF environment in contrast to the isolates from chronic infections that have been interpreted as the result of *P. aeruginosa* adaptation to CF airways. Depending on the early antibiotic treatment, infection, sooner or later, will evolve to chronic infection. The CF lung adaptation of *P. aeruginosa* is characterized by the transition from an acute environmental pathogen to a chronic CF-well-adapted pathogen, and the emergence of a phenotypically heterogeneous population. To establish chronic infections, *P. aeruginosa* loses some of its virulence potential (production of enzymes and toxins and lack of QS), slows down its growth rate, increases its antibiotic resistance (often multi-drug resistance), and/or reduces the stimulation of the immune system mainly due to a switch to the biofilm mode of growth, favored by overproduction of alginate. Typically, chronic *P. aeruginosa* isolates exhibited mucoid phenotype due to the overproduction of alginate and lack of flagella and pili. The presence of mutators in CF resident population is not strictly necessary to achieve adaptation, however, it represents a very large biological advantage in contrast with irreversible and accumulative mutations. Diversification of the metabolic pathways plays a fundamental role in the establishment of chronic infections in CF airways. In effect, *P. aeruginosa* can grow in microaerophilic and anaerobic zones by adjusting its metabolism. Denitrification, arginine fermentation and consumption of fatty acids are alternatives pathways to survive and growth in CF.

Future investigations should address the mechanisms underlying *P. aeruginosa* adaptation to CF airways to better understand them and to help design new therapeutic strategies. Studies about *P. aeruginosa* pathogenesis at early stages should be, especially, investigated more as the better way to avoid chronic infections is not allow their progression.
